# Impact of Protein Kinase C Activation and Monoclonal Antibodies on Immune Checkpoint Regulation and B Cell Function in Patients with Chronic Lymphocytic Leukemia

**DOI:** 10.3390/biomedicines13030741

**Published:** 2025-03-18

**Authors:** Aviwe Ntsethe, Phiwayinkosi Vusi Dludla, Bongani Brian Nkambule

**Affiliations:** 1 Department of Human Physiology, Nelson Mandela University, Gqeberha 6031, South Africa; aviwe.ntsethe@mandela.ac.za; 2 Department of Biochemistry and Microbiology, University of Zululand, KwaDlangezwa 3886, South Africa; dludlap@unizulu.ac.za; 3 School of Laboratory Medicine and Medical Sciences (SLMMS), College of Health Sciences, University of KwaZulu-Natal, Durban 4000, South Africa

**Keywords:** chronic lymphocytic leukemia, immune checkpoint inhibitor, B cell subsets, ionomycin, phorbol myristate acetate

## Abstract

**Background**: Chronic lymphocytic leukemia (CLL) is characterized by the proliferation of dysfunctional B cells, resulting in significant immune dysregulation. Patients with CLL exhibit varied responses to B cell receptor (BCR) targeted therapies, emphasizing the need for tailored immunotherapy approaches. This study investigated B cell function in untreated patients with CLL, and we further explored the effects of ex vivo protein kinase C activation on immune checkpoint expression and B cell profiles. **Methods:** Peripheral blood samples were collected from 21 untreated patients with CLL at King Edward Hospital in South Africa, between 2019 and 2022. B cells were stimulated with phorbol myristate acetate (PMA) and ionomycin. Using flow cytometry, the study explored the levels of B cell subsets and immune checkpoint proteins programmed cell death 1 (PD-1), programmed cell death-ligand 1 (PD-L1), programmed cell death-ligand 2 (PD-L2) and cytotoxic T-lymphocyte associated protein 4 (CTLA-4) expression on various B cell subsets. **Results:** PMA and ionomycin B cell stimulation upregulated PD-1, CTLA-4 and PD-L2 expression on B cell subsets (*p* < 0.01). As expected, monoclonal antibodies targeting PD-1, PD-L1 and CTLA-4 significantly downregulated the CTLA-4 expression of B cell subsets (*p* < 0.05), while PD-L2 exhibited varied responses in different B cell subsets. Moreover, PD-1 and PD-L1 expression on total B cells significantly declined following their blockage (*p* < 0.01). In addition, these monoclonal antibodies increased the levels of CD19^+^CD27^+^ B cells (*p* < 0.0128) and activated CD19^+^CD27^+^ B cells (*p* < 0.01). **Conclusions:** Protein kinase C activation on B cells stimulates immune checkpoint expression. The use of monoclonal antibodies on B cells plays a critical role in the B cell function through the reduction in CD38 expressing activated B cells and upregulation of CD19^+^CD27^+^ B cells. Moreover, the monoclonal antibody targeting PD-1, PD-L1 and CTLA-4 are effective in reducing the expression of CTLA-4 on B cell subsets, while PD-1 and PD-L1 blockage may be effective in reducing the expression of these immune checkpoints on total B cells.

## 1. Introduction

Chronic lymphocytic leukemia (CLL) is a lymphoproliferative malignancy mediated through B cell receptor (BCR) signaling and resistance to apoptosis due to overexpression of B cell lymphoma 2 (Bcl-2), resulting in the accumulation of mature CD5^+^, CD19^+^, CD20^+^, CD23^+^ functionally incompetent B cells in peripheral blood, bone marrow, lymph nodes, and the spleen [1,2]. Aberrations in adaptive immune responses, are characteristic features of CLL and drive immune suppression from the early stages of the disease [3,4]. Consequently, immune dysfunction increases the risk of secondary malignancies and infections, which are the main contributors to morbidity and mortality in patients with CLL [5].

In patients with CLL, B cell function is altered [6,7]. The BCR signaling pathway is one of the key factors contributing to the anti-apoptotic responses of malignant B cells in patients with CLL [8,9]. These signals contribute to cell survival and proliferation of malignant B cells in CLL [10]. In addition, malignant B cells in patients with CLL express chemokine receptors like CXCR4, which bind to CXCL12 in the bone marrow microenvironment [11]. This interaction supports malignant B cell homing to the bone marrow and provides survival signals [12].

Although immunosuppression is a state that is already evident in the early stages of CLL, disease progression or poor response to therapy is a consequence of immune dysfunction [13]. It has in fact become apparent that understanding immune responses is a vital aspect of understanding disease pathogenesis, which could potentially lead to the discovery of novel immunotherapies [3]. Activated B cells secrete immune checkpoints such as programmed cell death protein 1 (PD-1) to maintain immune tolerance, to suppress pathological autoimmune and inflammatory immune responses [14]. In CLL, BCR signaling promotes immune evasion by modulating the expression of immune checkpoints such as PD-1 and CTLA-4, suppressing T cell responses [3]. Immune checkpoint inhibition using monoclonal antibodies (mAbs) that target the cytotoxic T lymphocyte-associated protein 4 (CTLA-4) or PD-1 pathway has reshaped the landscape of therapeutic strategies for patients with CLL. Pembrolizumab, a PD-1-targeting agent, is associated with improved overall survival in patients with CLL [15]. However, a recent study, showed that patients with CLL may have variable treatment responses to CTLA-4 inhibitors, with unfavorable treatment outcomes in patients with high CTLA-4 expression [16]. CTLA-4 can regulate B cell activation both inside and outside of the germinal center, and this regulation can occur independently of CD80 and CD86 [17]. Therefore, CTLA-4’s role in inhibiting B cell activation still needs to be studied.

In patients with CLL, this BCR signaling pathway is disrupted [18,19]. Phorbol 12-myristate 13-acetate (PMA) activates the protein kinase C (PKC) pathway, specifically the classical PKC isoforms (PKCα, PKCβ, and PKCγ) by mimicking the action of diacylglycerol (DAG), a second messenger generated during BCR signaling [20]. PKC-β is immediately downstream of BCR and plays a vital role in malignant cell survival [21]. Understanding the modulation of immune checkpoints when PKC is activated can reveal whether these checkpoints contribute to survival or immune evasion in CLL. While PKC activation has been studied in relation to BCR signaling, its direct influence on immune checkpoint regulation in B cells has not been well explored. Furthermore, by modeling immune checkpoint blockage under different immunological activation and exhaustion settings we may stratify patients with CLL who will benefit from immunotherapy. We hypothesized that B cell stimulation would increase the levels of immune checkpoint expression on B cell subsets. The aim of this study was to investigate B cell function in untreated patients with CLL and to further explore the effects of ex vivo protein kinase C activation on immune checkpoint expression and B cell profiles.

## 2. Methods and Materials

### 2.1. Patient Recruitment

We recruited patients at King Edward VIII Hospital, a tertiary healthcare institution in Durban, KwaZulu-Natal, South Africa from July 2019 and May 2022. Ethical approval was obtained from the University of KwaZulu-Natal Biomedical Research Ethics Committee (BE456/18), South Africa. The study was conducted in accordance with the Declaration of Helsinki, and all participants provided written informed consent.

### 2.2. Inclusion and Exclusion Criteria

We included untreated patients with CLL along with age-matched healthy controls with no clinical signs of infection. Treated patients with CLL were excluded. Based on the treatment initiation criteria, 16 patients were on a “watch and wait” approach and 5 had a treatment indication at the time of sample collection [22].

### 2.3. Sample Collection

In total, 5 mL of peripheral blood samples were collected from consenting participants through venipuncture, using 6 mL ethylenediaminetetraacetic acid (EDTA) tubes (BD Bioscience, San Jose, CA, USA). Subsequently, the samples were transported under room temperature conditions (20–25 °C) from the hospital to the laboratory.

### 2.4. Isolation of Peripheral Blood Mononuclear Cells (PBMCs)

Peripheral blood mononuclear cells (PBMCs) were isolated from whole blood samples using the density-gradient centrifugation method with the use of Ficoll-Paque PLUS (Amersham, Biosciences, Uppsala, Sweden), as previously described [23]. Briefly, 3 mL of Ficoll-Paque PLUS was aliquoted into a 15 mL centrifuge tube (Sigma-Aldrich, Roedermark, Germany), followed by the careful layering of 4 mL of whole blood onto the Ficoll-Paque PLUS gradient. This was followed by subsequent centrifugation at 400× *g* for 40 min at 20 °C, the collected PBMCs was stored at −80 °C.

### 2.5. T Cell Depletion and B Cell Isolation from Peripheral Blood Mononuclear Cells

To enrich the B cell population within the obtained PBMCs, T cell depletion and positive B cell selection were conducted using the BD IMag isolation system (BD Bioscience, USA). Briefly, 50 µL of PBMCs were incubated with 5 µL of biotinylated human T lymphocyte enrichment cocktail (BD Biosciences, San Jose, CA, USA) for 15 min at room temperature. Following the incubation, 50 µL of streptavidin particles were introduced to the T cell-depleted PBMC samples and incubated for 30 min at room temperature. The samples were then reconstituted into 1 mL of 3.2% sodium-citrated buffer and placed on the BD IMag for 8 min. Isolated B cells were reconstituted into 100 µL phosphate-buffered saline (PBS) and stored at −80 °C.

### 2.6. Stimulation and Inhibition Assay

To assess B cell function in patients with CLL, B cells were treated with anti-CTLA-4, anti-PD-L1 and anti-PD-1 monoclonal antibodies at a concentration of 10 µg/mL for an hour [24]. We subsequently stimulated B cells as previously described [25,26]. Briefly, B cells were suspended in a complete medium [RPMI 1640 supplemented with 5% foetal bovine serum (FBS) and 1% liquid penicillin/streptomyosin (Biowest, Bradenton, FL, USA)] and stimulated with phorbol 12-myristate 13-acetate (PMA) (12.5 ng/mL; Cayman Chemical, Ann Arbor, MI, USA) and ionomycin (1.25 μg/mL; Cayman Chemical, Ann Arbor, MI, USA) for 12h, at 37 °C with 5% CO_2_. For the stimulation and inhibition assays, the following co-culture conditions were used; (1) PMA and ionomycin stimulated B cells; (2) PMA and ionomycin stimulated B cells plus anti-PD-1; (3) PMA and ionomycin stimulated B cells plus anti-PD-L1; (4) PMA and ionomycin stimulated B cells plus anti-CTLA-4.

### 2.7. Measurements of B Cell Subsets

**Figure 1:**
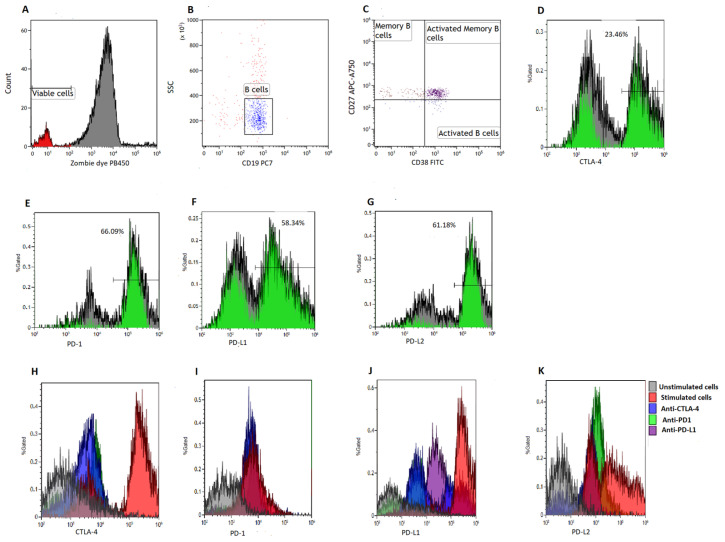
Gating strategy. B cells were isolated using magnetic bead sorting. (**A**) illustrates the gates applied to distinguish between viable and non-viable B cells based on a Zombie Aqua dye. (**B**) illustrates the gating on B cells based on side scatter (SSC) and CD19 expression. (**C**) illustrates the gating of activated B cells defined as CD19^+^CD38^+^CD27^−^ events, memory B cells defined as CD19^+^CD27^+^CD38^−^ events and activated memory B cells defined as CD19^+^CD27^+^CD38^+^ events, respectively. (**D**–**G**) illustrates the gating of CTLA-4, PD-1, PD-L1 and PD-L2, respectively. Histograms were used to demonstrate the levels of CTLA-4 (**H**), PD-1 (**I**), PD-L1 (**J**) and PD-L2 expression (**K**) on various co-culture conditions.

To quantify B cell subsets, we made use of a six-color flow cytometry panel consisting of CD38-FITC, CTLA-4-PE, PD-L2-APC, CD19-PE/Cy7, CD27-APC/Cy7, PD-1-PB450, PD-L1-PE and Zombie Aqua-BV421 (BioLegend, San Diego, CA, USA). Viable cells were defined as Zombie Aqua dye negative (Figure 1A). We acquired at least 5000 B cells (CD19^+^ events) (Figure 1B) and defined memory B cells (B_MEM_) as CD19^+^CD27^+^ events [27,28], activated B cells as CD19^+^CD27^−^CD38^+^ events and activated memory B cells as CD19^+^CD27^+^CD38^+^ events [28] (Figure 1C). Poststimulation B cell viability can be found in the Supplementary File (Supplementary Figure S1).

### 2.8. Measurements of Immune Checkpoint Levels on B Cell Subsets

To determine the levels of immune checkpoint expression on B cell subsets, we measured the expression of PD-1, PD-L1, PD-L2 and CTLA-4 on B_MEM_, and activated B cells (Figure 1D–G). A total of 5000 CD19^+^ events were acquired at a medium flow rate using the Beckman Coulter DxFLEX flow cytometer (Beckman Coulter, Inc., Brea, CA, USA) and analyzed using Kaluza version 1.2 (Beckman Coulter, Inc., Brea, CA, USA). Furthermore, we measured the levels of CTLA-4, PD-1, PD-L1 and PD-L2 expression on various co-culture conditions (Figure 1H–K).

### 2.9. Sample Size Estimation

We determined the minimum sample size of patients required to detect a large effect size (d) of 1.14 in the expression of immune checkpoints, at 80% power and alpha (α) of 0.05 using GPower 3.1.94 software (Universität, Düsseldorf, Germany). To detect a large effect size between two independent means using an unpaired *t*-test, we needed a minimum of twenty-one patients with CLL (n = 21) and twelve controls (n = 12).

### 2.10. Statistical Analysis

All statistical analysis was performed using GraphPad Prism version 8 software, (GraphPad Software Inc., San Diego, CA, USA). A repeated measures one-way ANOVA was used to compare parametric data with Dunnett’s as a post hoc test. The Friedman test was used to compare nonparametric data of the same group with Dunn’s as a post hoc test for multiple comparisons. To correct for multiple comparisons, a Bonferroni-corrected critical *p*-value of ˂0.0167 was considered statistically significant. Non-parametric data were reported as the median IQR and parametric data were presented as mean ± SD.

## 3. Results

### 3.1. Patients Characteristics

This study comprised 21 patients with CLL, with a mean age of 62.33 ± 13.31 years. The included study participants were multi-ethnic, and comprised of African (n = 19), Indian (n = 1) and European (n = 1). The study included 33.33% females and 66.67% males (Table 1).

### 3.2. Clinical Staging and Prognostic Markers in Patients with CLL

In patients with CLL, the diagnosis was based on standards from the International Workshop on Chronic Lymphocytic Leukaemia [1]. The clinical CLL stage was determined according to the Rai classification system [29], 47.61% were stage IV, 28.60% were stage III and 23.81% were stage II (Table 2). In our cohort, the most common cytogenetic abnormality was 11q22 deletion (33.33%), followed by 13q14 deletion (28.60%) and 17p13 deletion (14.30%). Only one patient had trisomy 12 (4.80%) and 19% had no abnormalities (Table 2).

### 3.3. Increased CD19^+^CD27^+^ B Cell Levels and a Reduction in Activated B Cells Following Immune Checkpoint Inhibition

**Figure 2:**
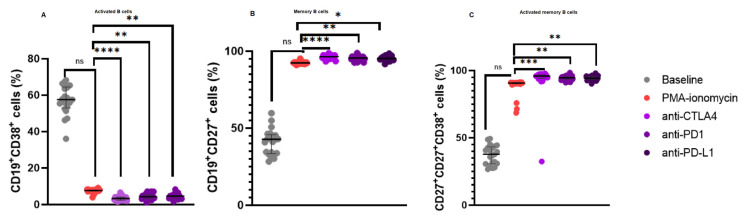
B Cell Subsets in patients with CLL upon B cell stimulation and immune checkpoint inhibition. (**A**) illustrates the expression levels of activated B cells, (**B**) memory B cells, and (**C**) activated memory B cells. The data are presented as the median ± interquartile range (IQR). *, **, *** and **** shows the level of significance between groups, ns: not significant.

In our B cell stimulation assays, there was no significant difference in the levels of activated B cells following PMA and ionomycin B cell stimulation 7.630% (8.060–7.350) when compared to the baseline levels 57.54% (64.57–52.97), *p* = 0.2480. However, there was a significant decrease in the levels of activated B cells following anti-CTLA-4 treatment 3.340% (4.085–2.410) when compared to PMA and ionomycin stimulated B cells 7.630% (8.060–7.350), *p* < 0.0001; anti-PD-1 treatment 4.300% (5.240–3.300) when compared to PMA and ionomycin stimulated B cells 7.630% (8.060–7.350), *p* = 0.0044; and anti-PD-L1 treatment 4.630% (4.940–3.505) when compared to PMA and ionomycin stimulated B cells 7.630% (8.060–7.350), *p* = 0.0044 (Figure 2A).

Furthermore, there was no significant difference in the levels of CD19^+^CD27^+^ B cells following PMA and ionomycin B cell stimulation 92.47% (92.71–91.99) when compared to the baseline levels 42.70% (45.76–33.40), *p* = 0.1917. However, there were significant increase in the levels of CD19^+^CD27^+^ B cells following anti-CTLA-4 treatment 96.48% (97.37–95.50) when compared to PMA and ionomycin stimulated B cells 92.47% (92.71–91.99), *p* < 0.0001; anti-PD-1 treatment 95.65% (96.38–94.79) when compared to PMA and ionomycin stimulated B cells 92.47% (92.71–91.99), *p* = 0.0064; and anti-PD-L1 treatment 95.30% (96.30–94.71) when compared to PMA and ionomycin stimulated B cells 92.47% (92.71–91.99), *p* = 0.0128 (Figure 2B).

Moreover, there was no significant difference in the levels of activated CD19^+^CD27^+^ B cells following PMA and ionomycin B cell stimulation 90.81% (91.11–90.17) when compared to the baseline levels 37.82% (43.21–30.74), *p* = 0.2480. However, there were significant increase in the levels of activated CD19^+^CD27^+^ B cells following anti-CTLA-4 treatment 95.99% (96.90–94.29) when compared to PMA and ionomycin stimulated B cells 90.81% (91.11–90.17), *p* = 0.0003; anti-PD-1 treatment 94.73% (95.45–93.43) when compared to PMA and ionomycin stimulated B cells 90.81% (91.11–90.17), *p* = 0.0044; and anti-PD-L1 treatment 94.41% (95.60–93.66) when compared to PMA and ionomycin stimulated B cells 90.81% (91.11–90.17), *p* = 0.0091 (Figure 2C).

### 3.4. Increased Levels of CTLA-4 Expression on B Cell Subsets Following B Cell Stimulation and Reduction Following Immune Checkpoint Inhibition

**Figure 3:**
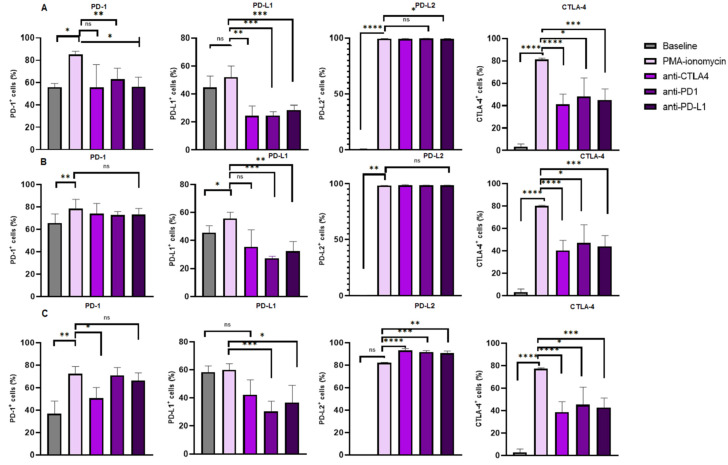
Immune checkpoint expression on B Cell subsets under different treatment conditions. The figure illustrates the levels of PD-L2 and CTLA-4 expression on (**A**) total B cells, (**B**) activated B cells and (**C**) memory B cells, respectively. The data are presented as the median ± interquartile range (IQR). *, **, *** and **** shows the level of significance between groups, ns: not significant.

The levels of CTLA-4 expression were significantly increased in the B cells following PMA and ionomycin B cell stimulation 81.49% (82.23–81.03) when compared to the baseline levels 3.270% (5.650–1.310), *p* < 0.0001. There was a significant decrease in the levels of CTLA-4 expression on B cells following anti-CTLA-4 treatment 41.09% (50.24–32.06) when compared to PMA and ionomycin stimulated B cells 81.49% (82.23–81.03), *p* < 0.0001; anti-PD-1 treatment 48.11% (64.42–41.25) when compared to PMA–ionomycin stimulated B cells 81.49% (82.23–81.03), *p* = 0.0341; and anti-PD-L1 treatment 44.92% (54.89–37.30) when compared to PMA and ionomycin stimulated B cells 81.49% (82.23–81.03), *p* = 0.0004 (Figure 3A).

Furthermore, levels of CTLA-4 expression were increased in activated B cells following PMA and ionomycin B cell stimulation 80.13% (80.75–79.75) when compared to the baseline levels 3.300% (5.650–1.310), *p* < 0.0001. However, the levels of CTLA-4 expression were decreased following anti-CTLA-4 treatment 40.36% (49.05–31.40) when compared to PMA and ionomycin stimulated B cells 80.13% (80.75–79.75), *p* < 0.0001; anti-PD-1 treatment 47.04% (63.21–40.26) when compared to PMA and ionomycin stimulated B cells 80.13% (80.75–79.75), *p* = 0.0341; and anti-PD-L1 treatment 44.13% (53.44–36.28) when compared to PMA and ionomycin stimulated B cells 80.13% (80.75–79.75), *p* = 0.0003 (Figure 3B).

Moreover, the levels of CTLA-4 expression were significantly increased in memory B cells following PMA and ionomycin B cell stimulation 77.28% (77.94–76.51) when compared to the baseline levels 2.880% (5.460–1.220), *p* < 0.0001. However, the levels of CTLA-4 expression were decreased following anti-CTLA-4 treatment 38.68% (47.68–30.36) when compared to PMA and ionomycin stimulated B cells 77.28% (77.94–76.51), *p* < 0.0001; anti-PD-1 treatment 45.03% (60.98–39.39) when compared to PMA and ionomycin stimulated B cells 77.28% (77.94–76.51), *p* = 0.0341; and anti-PD-L1 treatment 42.91% (51.01–35.33) when compared to PMA and ionomycin stimulated B cells 77.28% (77.94–76.51), *p* = 0.0004 (Figure 3C).

In summary, PMA and ionomycin B cell stimulation increase CTLA-4 surface expression on total B cells, activated B cells and memory B cells, while monoclonal antibodies targeting PD-1, PD-L1 and CTLA-4 significantly reduce the expression on these B cell subsets.

### 3.5. Increased Levels of PD-1 Expression on B Cell Subsets Following B Cell Stimulation

The levels of PD-1 expression were significantly increased in the B cells following PMA and ionomycin B cell stimulation (82.61 ± 7.960) when compared to the baseline levels (53.33% ± 16.18), *p* = 0.0132. However, the PD-1 expression levels on B cells following anti-PD-1 treatment (63.50% ± 9.042), *p* = 0.0168 and anti-PD-L1 treatment (56.18% ± 13.36), *p* = 0.0082 were significantly decreased when compared to that of the PMA and ionomycin stimulated B cells (82.61% ± 7.960). The PD-1 levels on B cells following anti-CTLA-4 (57.54% ± 21.27) were comparable to that of the PMA and ionomycin-stimulated B cells (82.61% ± 7.960), *p* = 0.0781 (Figure 3A).

Furthermore, levels of PD-1 expression were significantly increased in activated B cells following PMA and ionomycin B cell stimulation 78.41% (86.93–76.06) when compared to the baseline levels 65.68% (73.65–60.24), *p* = 0.0053. However, the levels of PD-1 expression on activated B cells following anti-CTLA-4 treatment 73.85% (83.17–63.84), *p* = 0.7029; anti-PD-1 treatment 72.62% (75.78–70.26), *p* = 0.3838 and anti-PD-L1 treatment 73.16% (78.56–67.25), *p* = 0.5327 were comparable to the PMA and ionomycin stimulated B cells 78.41% (86.93–76.06), Figure 3B.

In addition, the PD1 expression levels on memory B cells following PMA and ionomycin B cell stimulation 72.15% (78.82–67.66) were increased when compared to that of the baseline levels 36.85% (47.81–33.84), *p* = 0.0011. Interestingly, the PD-1 expression levels were significantly decreased following anti-CTLA-4 treatment 50.61% (60.22–42.84), *p* = 0.0185. However, these levels were comparable following anti-PD-1 treatment 70.76% (77.89–64.00), *p* > 0.9999; and anti-PD-L1 treatment 66.13% (72.97–53.97), *p* > 0.9999 when compared to PMA–ionomycin stimulated B cells 72.15% (78.82–67.66), Figure 3C.

In summary, PMA and ionomycin B cell stimulation increase PD-1 surface expression on total B cells, activated B cells and memory B cells, while monoclonal antibodies targeting PD-1 and PD-L1 significantly reduce the expression only on total B cells.

### 3.6. PD-L1 Expression Levels on B Cell Subsets Following B Cell Stimulation and Reduction Following Immune Checkpoint Inhibition

The PD-L1 expression levels on B cells following PMA and ionomycin B cell stimulation 52.00% (60.05–46.62) were comparable to the baseline levels 44.75% (52.71–31.23), *p* = 0.0881. However, PD-L1 expression levels were significant decrease on B cells following anti-CTLA-4 treatment 24.45% (31.52–16.97) when compared to PMA and ionomycin stimulated B cells 52.00% (60.05–46.62), *p* = 0.0066; anti-PD-1 treatment 24.56% (27.35–18.94) when compared to PMA–ionomycin stimulated B cells 52.00% (60.05–46.62), *p* = 0.0003; and anti-PD-L1 treatment 28.65% (31.96–24.09) when compared to PMA and ionomycin stimulated B cells 52.00% (60.05–46.62), *p* = 0.0002 (Figure 3A).

The PD-L1 expression levels on activated B cells following PMA and ionomycin B cell stimulation (51.71% ± 13.40) were increased when compared to the baseline levels (37.49% ± 17.29), *p* = 0.0231. However, these levels decreased following anti-PD-1 treatment (24.55% ± 5.647) when compared to PMA and ionomycin-stimulated B cells (51.71% ± 13.40), *p* = 0.0005 and anti-PD-L1 treatment (31.26% ± 8.056) when compared to PMA–ionomycin-stimulated B cells (51.71% ± 13.40), *p* = 0.0021. However, the levels were comparable following anti-CTLA-4 treatment (37.73% ± 12.88) when compared to PMA and ionomycin-stimulated B cells (51.71% ± 13.40), *p* = 0.0963 (Figure 3B).

Furthermore, the PD-L1 expression levels on memory B cells following PMA and ionomycin B cell stimulation 59.87% (64.40–55.31) were comparable when compared to the baseline levels 58.26% (62.75–46.22), *p* >0.9999. However, PD-L1 expression was decreased following anti-PD-1 treatment 30.35% (37.51–18.22) when compared to PMA and ionomycin stimulated B cells 59.87% (64.40–55.31), *p* = 0.0002; and anti-PD-L1 treatment 36.60% (49.13–26.59) when compared to PMA and ionomycin stimulated B cells 59.87% (64.40–55.31), *p* = 0.0298. However, PD-L1 expression was comparable following anti-CTLA-4 treatment 42.21% (52.65–30.92) when compared to PMA and ionomycin stimulated B cells 59.87% (64.40–55.31), *p* = 0.6599 (Figure 3C).

In summary, PMA and ionomycin B cell stimulation had no significant effect on PD-L1 surface expression on total B cells and memory B cells, while monoclonal antibodies targeting PD-1, PD-L1 and CTLA-4 significantly reduced the expression on these B cell subsets.

### 3.7. Increased Levels of PD-L2 Expression on B Cell Subsets Following B Cell Stimulation and Varied Expression Following Immune Checkpoint Inhibition

Levels of PD-L2 expression were significantly increased in B cells following PMA and ionomycin B cell stimulation (99.58% ± 0.06966) when compared to the baseline levels (0.3967% ± 0.3343), *p* < 0.0001. However, the expression of PD-L2 on B cells following anti-CTLA-4 treatment (99.33% ± 0.3552), *p* = 0.0502 and anti-PD-1 treatment (99.60% ± 0.2160), *p* = 0.9928 and anti-PD-L1 treatment (99.39% ± 0.2506), *p* = 0.0235 was comparable to that of the PMA and ionomycin stimulated B cells (99.58% ± 0.06966), Figure 3A.

Furthermore, levels of PD-L2 expression were significantly increased in activated B cells following PMA and ionomycin B cell stimulation (98.26% ± 0.1527) when compared to the baseline levels (0.3624% ± 0.2514), *p* < 0.0001. However, the levels of PD-L2 expression on activated B cells following anti-CTLA-4 treatment (98.55% ± 0.6834), *p* = 0.2811; anti-PD-1 treatment (98.53% ± 0.4412), *p* = 0.0639) and anti-PD-L1 treatment (98.41% ± 0.3380), *p* = 0.2955 were comparable to the PMA and ionomycin stimulated B cells (98.26% ± 0.1527), Figure 3B.

In addition, the levels of PD-L2 expression on memory B cells following PMA and ionomycin B cell stimulation 82.23% (82.58–81.44) were comparable to that of the baseline levels 0.2000% (0.2600–0.08000), *p* = 0.4042. Interestingly, the PD-L2 expression was significantly increased following anti-CTLA-4 treatment 92.97% (94.62–92.24), *p* < 0.0001; anti-PD-1 treatment 91.50% (92.86–89.31), *p* = 0.0006; and anti-PD-L1 treatment 90.66% (92.47–89.69), *p* = 0.0091 compared to PMA–ionomycin stimulated B cells 82.23% (82.58–81.44), Figure 3C.

In summary, PMA and ionomycin B cell stimulation increased PD-L2 surface expression on total B cells, activated B cells and memory B cells, while monoclonal antibodies targeting PD-1, PD-L1 and CTLA-4 had no effect on total and memory B cells and significantly increased the expression on memory B cells. The summary table showing the percentage expression of each immune checkpoint can be found in the Supplementary File (Tables S1–S3).

## 4. Discussion

The aim of this study was to investigate B cell function in untreated patients with CLL and to further explore the effects of ex vivo protein kinase C activation on immune checkpoint expression and B cell profiles. We previously evaluated the expression of immune checkpoints (PD-1 and CTLA-4) on B cells from patients with CLL as compared to healthy controls [30], we found that in patients with CLL, the expression of PD-1 and CTLA-4 were increased in activated B cells and memory B cells. In our current study, we observed increased levels of PD-1 and CTLA-4 expression on total B cells, activated B cells and memory B cells following PMA and ionomycin B cell stimulation. In addition, the PD-L2 protein was also elevated on total B cells and activated B cells. The PD-1 protein is one of the novel regulators of B cell activation [24]. The PD-1 receptor interacts with PD-L1 and PD-L2 which are released upon activation of B cells [31,32,33]. PD-L2 regulates antibody production through the inhibition of interleukin 5 (IL-5) production by T cells [34]. Elevated PD-L2 expression on activated B cells in patients with CLL may lead to low levels of plasma cells. Malignant B cells expressing CTLA-4 inhibit T cell activation [35]. Therefore, elevated CTLA-4 expression upon B cell activation in patients with CLL may lead to T cell exhaustion.

Immune checkpoints are important regulators of immune function and they prevent autoimmunity [36,37]. Malignant cells evade immune recognition and elimination partly due to the immunosuppression induced by upregulated immune checkpoints [38]. Monoclonal antibodies that target immune checkpoint molecules have been developed and have become the new standard therapy for numerous malignancies, including CLL [39,40,41]. Immune checkpoints such as PD-1 and CTLA-4 are dysregulated in patients with CLL [42]. As a result, immune checkpoint inhibitors, such as samalizumab and nivolumab targeting both B and T cell function, are becoming more effective as a CLL therapeutic strategy [43,44]. However, there are contradictory findings on the effectiveness of immune checkpoint inhibitors in patients with CLL [15,43,45,46,47].

In our study, we showed that blocking CTLA-4, PD-1 and PD-L1 upregulates the levels of CD19^+^CD27^+^ B cells and activated CD19^+^CD27^+^ B cells. In addition, our study showed that inhibition of these immune checkpoints downregulated the CD38-expressing activated B cells. The ability of the adaptive immune system to mount quick and efficient responses to infections depends on memory B cell development [48]. The accumulation of functional incompetent matured B cells, which characterizes CLL, disrupts the normal development and function of B cells, including memory B cells. Stimulation of the memory B cell development or activation could potentially enhance the immune response against malignant B cells [49,50]. Studies have shown that higher concentrations of memory B cells in patients with CLL are associated with good prognosis [50]. This may suggest that a higher percentage of memory B cells in patients with CLL could be associated with better clinical outcomes, including longer overall survival and slower disease progression. However, given that our study stimulated B cells for only 12 h, the observed increase in CD19^+^CD27^+^ B cells may not necessarily represent bona fide memory B cells. In addition, patients with CLL who have a higher proportion of malignant B cells expressing CD38 usually have a more aggressive form of the disease and a shorter overall survival [51,52]. The downregulation of activated B cells using immune checkpoint inhibitors may be beneficial for patients with CLL.

Our study demonstrated that anti-CTLA-4, anti-PD1 and anti-PD-L1 downregulate the expression of CTLA-4 on the total B cell population, activated B cells and memory B cells (Figure 3), whereas the expression of PD-L2 was upregulated on the CD19^+^CD27^+^ B cells following anti-CTLA-4, anti-PD1 and anti-PD-L1. A recent study [16] showed that CTLA-4 inhibition on CLL cell lines with an elevated expression of CTLA-4 stimulates the survival of malignant cells, whereas CTLA-4 inhibition in the low CTLA-4 expressing CLL cell lines does not affect apoptosis. This suggests that anti-CTLA therapy may be unfavorable in some patients with CLL. A plausible explanation for the increased PD-L2 expression may be that PD-L2 acts as a compensatory mechanism to counteract the effect of PD-L1 blockade [53]. Blocking PD-L1 may lead to compensatory upregulation of PD-L2, allowing malignant cells to maintain immune suppression. In addition, our study did not use monoclonal antibodies targeting PD-L2. To confirm this possibility, in our study, we demonstrated that anti-PD1 and anti-PD-L1 downregulate the expression of PD-L1 on total B cell population, activated B cells and memory B cells. Furthermore, PD-1 expression was downregulated on total B cells following this blockage.

The main limitation of this study is that monoclonal antibodies were only used as a monotherapy, we did not use a combination of these therapies. Dual immunotherapy has been shown to improve immune response and overall survival rate in patients with CLL [54,55,56]. Moreover, we did not determine the differences in cell death rates between activated and non-activated B cells. In addition, the antibody competition between the antibodies used for inhibition and those used for staining was not evaluated. Therefore, more studies to address these limitations are needed. Due to the low sample size in our study, the B cell-mediated immune response should be further investigated in larger patient cohorts, particularly in another Sub-Saharan African country to confirm these findings. Furthermore, we did not compare immune checkpoint expression between CLL and healthy B cells following PKC activation. While our previous work demonstrated elevated baseline immune checkpoint expression in CLL B cells compared to healthy controls, it remains unclear whether the changes observed after PKC activation are unique to CLL B cells. Future studies should explore this comparison to determine whether PKC-driven immune checkpoint modulation differs between malignant and normal B cells.

## 5. Conclusions

Protein kinase C activation on B cells stimulates immune checkpoint expression. The use of monoclonal antibodies on B cells plays a critical role in the B cell function through the reduction in CD38 expressing activated B cells and upregulation of CD19^+^CD27^+^ B cells. Moreover, the monoclonal antibody targeting PD-1, PD-L1 and CTLA-4 are effective in reducing the expression of CTLA-4 on B cell subsets, while PD-1 and PD-L1 blockage may be effective in reducing the expression of these immune checkpoints on total B cells.

## Figures and Tables

**Table 1:** The baseline characteristics and hematological parameters of the participants.

	Control (n = 12)	Patients with CLL [21]	*p*-Value
Age (Years)	56.58 ± 15.67	62.33 ± 13.31	0.2714
Male, n (%)	58.33	61.9	
Female, n (%)	41.67	38.1	
White blood cell count (10^3^ µL)	5.26 ± 1.38	130.4 ± 29.71	0.0005
Red blood cell (10^6^ µL)	4.74 ± 0.94	2.10 ± 0.84	<0.0001
Haemoglobin (g/dL)	14.13 ± 3.81	8.19 ± 2.30	<0.0001
Platelets (10^3^ µL)	210.4 ± 73.14	157.5 ± 141.9	0.1831

**Table 2:** Clinical staging and prognostic markers in patients with CLL (n = 21).

Clinical Parameters	
RAI Staging	
I, n (%)	0 (0)
II, n (%)	5 (23.8)
III, n (%)	6 (28.6)
IV, n (%)	10 (47.6)
FISH Status	
Trisomy 12, n (%)	1 (4.8)
Deletions	
11q22, n (%)	7 (33.3)
13q14, n (%)	6 (28.6)
17p13, n (%)	3 (14.3)
no abnormalities, n (%)	4 (19.0)
CLL-IPI	
Low risk, n (%)	14 (66.7)
Intermediate risk, n (%)	4 (19)
High risk, n (%)	3 (14.3)
Prognostic Biomarkers	
B2M mg/L	0.74 ± 0.30

## Data Availability

The original contributions presented in the study are included in the article and Supplementary Material, further inquiries can be directed to the corresponding author/s.

## Supplementary Materials

The following supporting information can be downloaded at: https://www.mdpi.com/article/10.3390/biomedicines13030741/s1, Figure S1: Post-stimulation viability. 83.6% viable B cells; Table S1: Immune checkpoints on total B cells; Table S2: Immune checkpoints on activated B cells; Table S3: Immune checkpoints on CD19^+^CD27^+^ cells.

## References

[1] Hallek M., Cheson B.D., Catovsky D., Caligaris-Cappio F., Dighiero G., Döhner H., Hillmen P., Keating M., Montserrat E., Chiorazzi N. (2018). iwCLL guidelines for diagnosis, indications for treatment, response assessment, and supportive management of CLL. Blood J. Am. Soc. Hematol..

[2] Munir T., Cairns D.A., Bloor A., Allsup D., Cwynarski K., Pettitt A., Paneesha S., Fox C.P., Eyre T.A., Forconi F. (2024). Chronic lymphocytic leukemia therapy guided by measurable residual disease. N. Engl. J. Med..

[3] Arruga F., Gyau B.B., Iannello A., Vitale N., Vaisitti T., Deaglio S. (2020). Immune response dysfunction in chronic lymphocytic leukemia: Dissecting molecular mechanisms and microenvironmental conditions. Int. J. Mol. Sci..

[4] Haseeb M., Anwar M.A., Choi S. (2018). Molecular interactions between innate and adaptive immune cells in chronic lymphocytic leukemia and their therapeutic implications. Front. Immunol..

[5] Teh B.W., Tam C.S., Handunnetti S., Worth L.J., Slavin M.A. (2018). Infections in patients with chronic lymphocytic leukaemia: Mitigating risk in the era of targeted therapies. Blood Rev..

[6] Karabon L., Partyka A., Ciszak L., Pawlak-Adamska E., Tomkiewicz A., Bojarska-Junak A., Roliński J., Wołowiec D., Wrobel T., Frydecka I. (2020). Abnormal expression of BTLA and CTLA-4 immune checkpoint molecules in chronic lymphocytic leukemia patients. J. Immunol. Res..

[7] Solman I.G., Blum L.K., Hoh H.Y., Kipps T.J., Burger J.A., Barrientos J.C., O’Brien S., Mulligan S.P., Kay N.E., Hillmen P. (2020). Ibrutinib restores immune cell numbers and function in first-line and relapsed/refractory chronic lymphocytic leukemia. Leuk. Res..

[8] Chen R., Tsai J., Thompson P.A., Chen Y., Xiong P., Liu C., Burrows F., Sivina M., Burger J.A., Keating M.J. (2021). The multi-kinase inhibitor TG02 induces apoptosis and blocks B-cell receptor signaling in chronic lymphocytic leukemia through dual mechanisms of action. Blood Cancer J..

[9] Ondrisova L., Mraz M. (2020). Genetic and non-genetic mechanisms of resistance to BCR signaling inhibitors in B cell malignancies. Front. Oncol..

[10] Pascutti M.F., Jak M., Tromp J.M., Derks I.A., Remmerswaal E.B., Thijssen R., van Attekum M.H., van Bochove G.G., Luijks D.M., Pals S.T. (2013). IL-21 and CD40L signals from autologous T cells can induce antigen-independent proliferation of CLL cells. Blood J. Am. Soc. Hematol..

[11] Burger J.A. (2010). Chemokines and chemokine receptors in chronic lymphocytic leukemia (CLL): From understanding the basics towards therapeutic targeting. Seminars in Cancer Biology.

[12] Mehrpouri M. (2022). The contributory roles of the CXCL12/CXCR4/CXCR7 axis in normal and malignant hematopoiesis: A possible therapeutic target in hematologic malignancies. Eur. J. Pharmacol..

[13] Morrison V.A. (2010). Infectious complications of chronic lymphocytic leukaemia: Pathogenesis, spectrum of infection, preventive approaches. Best Pract. Res. Clin. Haematol..

[14] Han Y., Liu D., Li L. (2020). PD-1/PD-L1 pathway: Current researches in cancer. Am. J. Cancer Res..

[15] Ding W., LaPlant B.R., Call T.G., Parikh S.A., Leis J.F., He R., Shanafelt T.D., Sinha S., Le-Rademacher J., Feldman A.L. (2017). Pembrolizumab in patients with CLL and Richter transformation or with relapsed CLL. Blood J. Am. Soc. Hematol..

[16] Ciszak L., Frydecka I., Wolowiec D., Szteblich A., Kosmaczewska A. (2016). Patients with chronic lymphocytic leukaemia (CLL) differ in the pattern of CTLA-4 expression on CLL cells: The possible implications for immunotherapy with CTLA-4 blocking antibody. Tumor Biol..

[17] Sage P.T., Paterson A.M., Lovitch S.B., Sharpe A.H. (2014). The coinhibitory receptor CTLA-4 controls B cell responses by modulating T follicular helper, T follicular regulatory, and T regulatory cells. Immunity.

[18] Smith L.D., Minton A.R., Blunt M.D., Karydis L.I., Dutton D.A., Rogers-Broadway K.-R., Dobson R., Liu R., Norster F., Hogg E. (2020). BCR signaling contributes to autophagy regulation in chronic lymphocytic leukemia. Leukemia.

[19] Skånland S.S., Karlsen L., Taskén K. (2020). B cell signalling pathways—New targets for precision medicine in chronic lymphocytic leukaemia. Scand. J. Immunol..

[20] Griner E.M., Kazanietz M.G. (2007). Protein kinase C and other diacylglycerol effectors in cancer. Nat. Rev. Cancer.

[21] El-Gamal D., Williams K., LaFollette T.D., Cannon M., Blachly J.S., Zhong Y., Woyach J.A., Williams E., Awan F.T., Jones J. (2014). PKC-β as a therapeutic target in CLL: PKC inhibitor AEB071 demonstrates preclinical activity in CLL. Blood J. Am. Soc. Hematol..

[22] Hallek M., Cheson B.D., Catovsky D., Caligaris-Cappio F., Dighiero G., Döhner H., Hillmen P., Keating M.J., Montserrat E., Rai K.R. (2008). Guidelines for the diagnosis and treatment of chronic lymphocytic leukemia: A report from the International Workshop on Chronic Lymphocytic Leukemia updating the National Cancer Institute–Working Group 1996 guidelines. Blood J. Am. Soc. Hematol..

[23] Jaatinen T., Laine J. (2007). Isolation of Mononuclear Cells from Human Cord Blood by Ficoll-Paque Density Gradient. Curr. Protoc. Stem Cell Biol..

[24] Thibult M.-L., Mamessier E., Gertner-Dardenne J., Pastor S., Just-Landi S., Xerri L., Chetaille B., Olive D. (2013). PD-1 is a novel regulator of human B-cell activation. Int. Immunol..

[25] Bouaziz J.D., Calbo S., Maho-Vaillant M., Saussine A., Bagot M., Bensussan A., Musette P. (2010). IL-10 produced by activated human B cells regulates CD4+ T-cell activation in vitro. Eur. J. Immunol..

[26] Van Hoof D., Lomas W., Hanley M.B., Park E. (2014). Simultaneous flow cytometric analysis of IFN-γ and CD4 mRNA and protein expression kinetics in human peripheral blood mononuclear cells during activation. Cytom. Part A.

[27] Axelsson S., Magnuson A., Lange A., Alshamari A., Hörnquist E.H., Hultgren O. (2020). A combination of the activation marker CD86 and the immune checkpoint marker B and T lymphocyte attenuator (BTLA) indicates a putative permissive activation state of B cell subtypes in healthy blood donors independent of age and sex. BMC Immunol..

[28] Joscelyn J., Ochoa-Repáraz J., Kasper L. (2020). Principles of Immunotherapy. Clinical Neuroimmunology.

[29] Rai K.R., Sawitsky A., Cronkite E.P., Chanana A.D., Levy R.N., Pasternack B.S. (1975). Clinical staging of chronic lymphocytic leukemia. Blood.

[30] Ntsethe A., Mkhwanazi Z.A., Dludla P.V., Nkambule B.B. (2024). B Cell Subsets and Immune Checkpoint Expression in Patients with Chronic Lymphocytic Leukemia. Curr. Issues Mol. Biol..

[31] Latchman Y., Wood C.R., Chernova T., Chaudhary D., Borde M., Chernova I., Iwai Y., Long A.J., Brown J.A., Nunes R. (2001). PD-L2 is a second ligand for PD-1 and inhibits T cell activation. Nat. Immunol..

[32] Kaku H., Rothstein T.L. (2010). Octamer binding protein 2 (Oct2) regulates PD-L2 gene expression in B-1 cells through lineage-specific activity of a unique, intronic promoter. Genes Immun..

[33] Yamazaki T., Akiba H., Iwai H., Matsuda H., Aoki M., Tanno Y., Shin T., Tsuchiya H., Pardoll D.M., Okumura K. (2002). Expression of programmed death 1 ligands by murine T cells and APC. J. Immunol..

[34] McKay J.T., Haro M.A., Daly C.A., Yammani R.D., Pang B., Swords W.E., Haas K.M. (2017). PD-L2 Regulates B-1 Cell Antibody Production against Phosphorylcholine through an IL-5-Dependent Mechanism. J. Immunol..

[35] Do P., Beckwith K.A., Cheney C., Tran M., Beaver L., Griffin B.G., Mo X., Liu Y., Lapalombella R., Hertlein E. (2019). Leukemic B Cell CTLA-4 Suppresses Costimulation of T Cells. J. Immunol..

[36] Hogg S.J., Vervoort S.J., Deswal S., Ott C.J., Li J., Cluse L.A., Beavis P.A., Darcy P.K., Martin B.P., Spencer A. (2017). BET-bromodomain inhibitors engage the host immune system and regulate expression of the immune checkpoint ligand PD-L1. Cell Rep..

[37] Haanen J., Ernstoff M., Wang Y., Menzies A., Puzanov I., Grivas P., Larkin J., Peters S., Thompson J., Obeid M. (2020). Autoimmune diseases and immune-checkpoint inhibitors for cancer therapy: Review of the literature and personalized risk-based prevention strategy. Ann. Oncol..

[38] Marin-Acevedo J.A., Kimbrough E.O., Lou Y. (2021). Next generation of immune checkpoint inhibitors and beyond. J. Hematol. Oncol..

[39] Hillmen P., Robak T., Janssens A., Babu K.G., Kloczko J., Grosicki S., Doubek M., Panagiotidis P., Kimby E., Schuh A. (2015). Chlorambucil plus ofatumumab versus chlorambucil alone in previously untreated patients with chronic lymphocytic leukaemia (COMPLEMENT 1): A randomised, multicentre, open-label phase 3 trial. Lancet.

[40] Seymour J.F., Kipps T.J., Eichhorst B., Hillmen P., D’Rozario J., Assouline S., Owen C., Gerecitano J., Robak T., De la Serna J. (2018). Venetoclax–rituximab in relapsed or refractory chronic lymphocytic leukemia. N. Engl. J. Med..

[41] Hallek M. (2023). First line therapy of CLL. Hematol. Oncol..

[42] Palma M., Gentilcore G., Heimersson K., Mozaffari F., Näsman-Glaser B., Young E., Rosenquist R., Hansson L., Österborg A., Mellstedt H. (2017). T cells in chronic lymphocytic leukemia display dysregulated expression of immune checkpoints and activation markers. Haematologica.

[43] Younes A., Brody J., Carpio C., Lopez-Guillermo A., Ben-Yehuda D., Ferhanoglu B., Nagler A., Ozcan M., Avivi I., Bosch F. (2019). Safety and activity of ibrutinib in combination with nivolumab in patients with relapsed non-Hodgkin lymphoma or chronic lymphocytic leukaemia: A phase 1/2a study. Lancet Haematol..

[44] Mahadevan D., Lanasa M.C., Farber C., Pandey M., Whelden M., Faas S.J., Ulery T., Kukreja A., Li L., Bedrosian C.L. (2019). Phase I study of samalizumab in chronic lymphocytic leukemia and multiple myeloma: Blockade of the immune checkpoint CD200. J. Immunother. Cancer.

[45] Jain N., Basu S., Thompson P.A., Ohanian M., Ferrajoli A., Pemmaraju N., Cortes J.E., Estrov Z., Burger J.A., Neelapu S.S. (2016). Nivolumab combined with ibrutinib for CLL and Richter transformation: A phase II trial. Blood.

[46] Archibald W.J., Meacham P.J., Williams A.M., Baran A.M., Victor A.I., Barr P.M., Sahasrahbudhe D.M., Zent C.S. (2018). Management of melanoma in patients with chronic lymphocytic leukemia. Leuk. Res..

[47] Mato A., Svoboda J., Luning Prak E., Schuster S., Tsao P., Dorsey C., Sarmasti L., Becker P., Brander D., Nasta S. (2019). Phase I/II study of umbralisib (TGR-1202) in combination with ublituximab (TG-1101) and pembrolizumab in patients with Rel/Ref CLL and Richter’s transformation. Hematol. Oncol..

[48] Akkaya M., Kwak K., Pierce S.K. (2020). B cell memory: Building two walls of protection against pathogens. Nat. Rev. Immunol..

[49] Mékinian A., Quinquenel A., Belkacem K.A., Kanoun F., Dondi E., Franck E., Boubaya M., Mhibik M., Baran-Marszak F., Letestu R. (2023). Immuno-regulatory malignant B cells contribute to Chronic Lymphocytic Leukemia progression. Cancer Gene Ther..

[50] Awan F.T., Byrd J.C., Niederhuber J.E., Armitage J.O., Kastan M.B., Doroshow J.H., Tepper J.E. (2020). 99—Chronic Lymphocytic Leukemia. Abeloff’s Clinical Oncology.

[51] Malavasi F., Deaglio S., Damle R., Cutrona G., Ferrarini M., Chiorazzi N. (2011). CD38 and chronic lymphocytic leukemia: A decade later. Blood J. Am. Soc. Hematol..

[52] Manna A., Aulakh S., Jani P., Ahmed S., Akhtar S., Coignet M., Heckman M., Meghji Z., Bhatia K., Sharma A. (2019). Targeting CD38 Enhances the Antileukemic Activity of Ibrutinib in Chronic Lymphocytic Leukemia. Clin. Cancer Res..

[53] Bodhankar S., Galipeau D., Vandenbark A.A., Offner H. (2013). PD-1 Interaction with PD-L1 but not PD-L2 on B-cells Mediates Protective Effects of Estrogen against EAE. J. Clin. Cell. Immunol..

[54] Al-Sawaf O., Zhang C., Tandon M., Sinha A., Fink A.-M., Robrecht S., Samoylova O., Liberati A.M., Pinilla-Ibarz J., Opat S. (2020). Venetoclax plus obinutuzumab versus chlorambucil plus obinutuzumab for previously untreated chronic lymphocytic leukaemia (CLL14): Follow-up results from a multicentre, open-label, randomised, phase 3 trial. Lancet Oncol..

[55] Moreno C., Greil R., Demirkan F., Tedeschi A., Anz B., Larratt L., Simkovic M., Samoilova O., Novak J., Ben-Yehuda D. (2019). Ibrutinib plus obinutuzumab versus chlorambucil plus obinutuzumab in first-line treatment of chronic lymphocytic leukaemia (iLLUMINATE): A multicentre, randomised, open-label, phase 3 trial. Lancet Oncol..

[56] Shanafelt T.D., Wang X.V., Kay N.E., Hanson C.A., O’Brien S., Barrientos J., Jelinek D.F., Braggio E., Leis J.F., Zhang C.C. (2019). Ibrutinib–rituximab or chemoimmunotherapy for chronic lymphocytic leukemia. N. Engl. J. Med..

