# Supplementary material for: Impact of Protein Kinase C Activation and Monoclonal Antibodies on Immune Checkpoint Regulation and B Cell Function in Patients with Chronic Lymphocytic Leukemia
Source: Biomedicines. 2025 Mar 18;13(3):741. doi: 10.3390/biomedicines13030741 (PMC11940456; doi:10.3390/biomedicines13030741)
Supplement: Supplementary file 1 [file biomedicines-13-00741-s001.zip › Supplementary 2 IC percentages.pdf]

Immune checkpoints reported as mean percentage± standard deviation (Table 1-3).

Table S1: Immune checkpoints on total B cells

	Unstimulated cells	Stimulated cells	anti-PD-1 treated cells	anti-PD-L1 treated cells	anti-CTLA-4 treated cells
PD-1	53.33± 16.18	82.61± 7.960	63.50± 9.042	56.18± 13.36	57.54± 21.27
PD-L1	39.87± 17.02	51.23± 11.97	23.35± 4.664	28.25± 5.343	25.98± 11.81
PD-L2	0.3967± 0.3343	99.58± 0.0696	99.60± 0.2160	99.39± 0.2506	99.33± 0.3552
CTLA-4	3.630± 2.897	82.35± 3.868	52.73± 13.97	44.87± 10.05	41.93± 11.32

Table S2: Immune checkpoints on activated B cells

	Unstimulated cells	Stimulated cells	anti-PD-1 treated cells	anti-PD-L1 treated cells	anti-CTLA-4 treated cells
PD-1	63.77± 19.69	81.00± 7.276	72.92± 4.654	73.03± 6.472	73.67± 12.76
PD-L1	37.49± 17.29	51.71± 13.40	24.55± 5.647	31.26± 8.056	37.73± 12.88
PD-L2	0.3624± 0.2514	98.26± 0.1527	98.53± 0.4412	98.41± 0.3380	98.55± 0.6834
CTLA-4	3.631± 2.896	80.99± 4.012	51.59± 13.95	43.75± 9.802	41.00± 10.86

Table S3: Immune checkpoints on $CD19^+CD27^+$ cells

	Unstimulated cells	Stimulated cells	anti-PD-1 treated cells	anti-PD-L1 treated cells	anti-CTLA-4 treated cells
PD-1	43.50± 18.11	73.40± 7.615	69.87± 9.485	64.99± 12.62	48.76± 17.64
PD-L1	50.55± 19.59	56.08± 14.11	28.26± 8.960	37.29± 11.08	42.46± 15.04
PD-L2	0.2005± 0.1297	82.14± 1.579	91.39± 2.746	90.87± 2.505	92.95± 2.115
CTLA-4	3.439± 2.767	79.03± 4.140	50.23± 13.68	42.48± 9.420	39.87± 10.62
