# Supplementary figures and images for: Impact of Protein Kinase C Activation and Monoclonal Antibodies on Immune Checkpoint Regulation and B Cell Function in Patients with Chronic Lymphocytic Leukemia
Source: Biomedicines. 2025 Mar 18;13(3):741. doi: 10.3390/biomedicines13030741 (PMC11940456; doi:10.3390/biomedicines13030741)

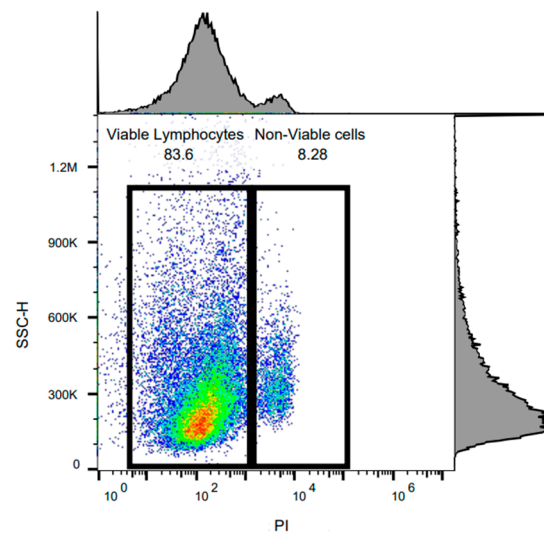

**Supplementary Figure S1:** Post-stimulation viability. 83.6% viable B cells.

Supplement: Supplementary file 1 [file biomedicines-13-00741-s001.zip › Supplementary 1.pdf]
